# Antiprotozoal and Antimycobacterial Activities of Pure Compounds from *Aristolochia elegans* Rhizomes

**DOI:** 10.1155/2012/593403

**Published:** 2012-02-16

**Authors:** Adelina Jiménez-Arellanes, Rosalba León-Díaz, Mariana Meckes, Amparo Tapia, Gloria María Molina-Salinas, Julieta Luna-Herrera, Lilián Yépez-Mulia

**Affiliations:** ^1^Unidad de Investigación Médica en Farmacología de Productos Naturales (UIMFPN), UMAE Hospital de Pediatría, Centro Médico Nacional Siglo XXI (CMN-SXXI), Instituto Mexicano del Seguro Social, (IMSS), 2° piso CORSE, 06720 México, DF, Mexico; ^2^Unidad de Investigación Médica en Enfermedades Infecciosas y Parasitarias (UIMEIP), UMAE Hospital de Pediatría, CMN-SXXI, IMSS, 2° piso, 06720 México, DF, Mexico; ^3^División de Biología Celular y Molecular, Centro de Investigación Biomédica de Noreste (CIBIN), IMSS, 64720 Monterrey, NL, Mexico; ^4^Laboratorio de Inmunoquímica II, Departamento de Inmunoquímica, Escuela Nacional de Ciencias Biológicas, Instituto Politécnico Nacional, 11340 México, DF, Mexico

## Abstract

We analyzed the antimycobacterial activity of the hexane extract of rhizomes from *Aristolochia elegans*. Some compounds of this extract were purified and tested against a group of drug-resistant *Mycobacterium tuberculosis* strains. We also evaluated their antiprotozoal activities. The hexane extract was active against *M. tuberculosis* H37Rv at a MIC = 100 *μ*g mL^−1^; the pure compounds eupomatenoid-1, fargesin, and (8R,8′R,9R)-cubebin were active against *M. tuberculosis* H37Rv (MIC = 50 *μ*g mL^−1^), while fargesin presented activity against three monoresistant strains of *M. tuberculosis* H37Rv and a MDR clinical isolate of *M. tuberculosis* (MIC < 50 *μ*g mL^−1^). Both the extract and eupomatenoid-1 were very active against *E. histolytica* and *G. lamblia* (IC_50_ < 0.624 *μ*g mL^−1^); in contrast, fargesin and (8R,8′R,9R)-cubebin were moderately active (IC_50_ < 275 *μ*g mL^−1^). In this context, two compounds responsible for the antimycobacterial presented by *A. elegans* are fargesin and cubebin, although others may exert this activity also. In addition to the antimycobacterial activity, the hexane extract has important activity against *E. histolytica* and *G. lamblia*, and eupomatenoid-1 is one of the compounds responsible for the antiparasite activity.

## 1. Introduction

*Aristolochia elegans* Mast (Aristolochiaceae) syn. *A. littoralis* is commonly known as guaco, duck flower, or elephant foot and is a perennial shrub cultivated as an ornamental plant in several parts of the world [1, 2]. The genus *Aristolochia* comprises ca. 400 species and is distributed in wide areas from tropical to template zones [3]. On the American continent, it is found from the south of the USA, throughout Mexico, the Caribbean, and Central America and as far as Argentina [4, 5]. *A. elegans* has been employed as an expectorant, an antitussive, an antiasthmatic, an analgesic, an antihistamine, and a detoxicant agent [3]. Moreover, *A. elegans* is utilized as an antidote against snake bites and toothache, as a purgative, an insecticide, and as an antispasmodic [6]. In Mexican traditional medicine, this plant is used as antimicrobial, antitumoral, antidiarrheal, antipyretic, emmenagic agent, and anti-snake venom and for the treatment of scorpion poisoning [6, 7]. Alkaloids, lignans, neolignans, monoterpenoids, diterpenoids, sesquiterpenoids, tetralones, isoquinolines, porphyrins, biphenyl ethers, aristolactolactams, and aristolochic acid dimers have been isolated from the organic extracts or essential oil of leaves, stems, and roots of this species [2–5]. The hexane (Hex) and methanol (MeOH) extracts of *A. elegans *have proven to be moderately active against the venom of *Centruroides limpidus limpidus*, and the mixture of hexanic extracts from *A. elegans* and *Bouvardia ternifolia* has improved their inhibitory effects up to 70% [6]. On the other hand, *A. elegans* ethanolic (EtOH) extract exhibited antimitotic and antiviral activities [3, 8]. In a preliminary study, we focused on the analysis the activity of the Hex and MeOH extract (at 100 *μ*g mL^−1^) from the leaves, seeds, and rhizomes of *A. elegans* against *M. tuberculosis* H37Rv by radiorespirometric Bactec 460 assay. The Hex extract from leaves and seeds reduced the mycobacterium growth by less than 70%; however, with the Hex extract from the rhizome, a 99% inhibition of *M. tuberculosis* H37Rv growth was reached (data no published). Based on these data, we decided to investigate the antimycobacterial activity of the major compounds found in the Hex extract of *A. elegans-*rhizome.

In this paper, the isolation of (8R,8′R,9R)-cubebin, fargesin, and eupomatenoid-1 from the active Hex extract of *A. elegans* rhizome is described and their antimycobacterial activity against four monoresistant and two MDR *M. tuberculosis* strains is demonstrated. In addition, the activity of the isolated compounds was tested against the anaerobic protozoa: *Entamoeba histolytica* and *Giardia lamblia*.

## 2. Methods

### 2.1. General Experimental Procedures

The chemical characterization of the isolated compounds was determined by ^1^H-NMR (Bruker-Avance F, 300 MHz) and ^13^C-NMR (Variant Unity, 75.4 MHz) using Tetramethylsilane as an internal standard in CDCl_3_. Electron impact-mass spectra (EI-MS) were obtained on a Jeol AX-505 HA mass spectrometer at 70 eV. Melting points (m.p.) were determined with a Fisher-Johns apparatus and are uncorrected. Open Column chromatography (CC) was carried out by using silica gel 60 GF_254_ (70–230 mesh, Merck) as a stationary phase, and silica gel 60 F_254_ precoated aluminum plates (0.2 mm, Merck) were employed for analytical and preparative Thin Layer Chromatography (TLC) analysis. Hex, chloroform (CHCl_3_), and MeOH were obtained from Mallinckrodt and J. T. Baker.

The spots were visualized by spraying it with a 10% solution of aqueous H_2_SO_4_ followed by heating at 100°C. High Performance Liquid Chromatography (HPLC) analyses were carried out with a Waters 600 system controller connected to a photodiode array detector 996, which was programmed to collect data from 220–380 nm at 2.4-nm resolutions. Control of equipment, data acquisition, and processing and the management of chromatographic information were performed by Millennium 32 software program (Waters). Analyses were accomplished on a Spherisorb S100DS2 RP column (4.6 × 250 mm, 10-*μ*m particle size, Waters). The mobile phase comprised an isocratic MeOH system (HPLC grade, J. T. Baker), except for eupomatenoid-1 whose mobile phase was composed of acetonitrile/formic acid 98 : 2 (both HPLC grade, J. T. Baker). The flow rate was maintained constant at 0.3 mL min^−1^ for 30 min. Samples were solubilized in MeOH at 1 mg mL^−1^, and a volume of 20 *μ*L was injected.

### 2.2. Plant Material

*Aristolochia elegans*-rhizome was collected in Miahuatlan, Oaxaca State, Mexico, in November 2006. The plant was botanically identified by Abigail Aguilar, M.Sc., and a voucher specimen was deposited at the Herbarium of the Instituto Mexicano del Seguro Social, Mexico (IMSSM) with code number 16080.

### 2.3. Extraction and Isolation

Powdered air-dried rhizome (530 g) was macerated (3 × 48 h) with 5 L Hex at room temperature. The extract obtained was filtered and vacuum concentrated to yield 37 g of the crude extract. The Hex extract (35 g) was subjected to CC in silica gel (150 g) and was eluted with Hex : CHCl_3_ (100→0) and CHCl_3_ : MeOH (100→0), and 171 fractions of 125 mL each were obtained. Primary fractions (F1–F15) were combined according to a TLC analysis as follows: F1 (69 mg); F2 (10 mg); F3 (18 mg); F4 (92 mg); F5 (69 mg); F6 (149 mg); F7 (115 mg); F8 (434 mg); F9 (258 mg); F10 (322 mg); F11 (1,816 mg); F12 (1,218 mg); F13 (669 mg); F14 (14,109 mg); F15 (5,870 mg).

Fraction F5–F10 was submitted to preparative TLC employing Hex : CHCl_3_ 70 : 30 as an elution system; after this procedure, 53.5 mg of eupomatenoid-1 (**1**) was obtained with *R_f_* = 0.13. On the other hand, primary fraction F14 (13 g) was subjected to repeated CC, utilizing silica gel (75 g) with solvent gradients of Hex : CHCl_3 _(100 to 0) and CHCl_3_ : MeOH (100 to 0). This process yielded 13 secondary fractions (FA-FM) of 150 mL each as follows: FA (9 mg); FB (11 mg); FC (69 mg); FD (10 mg); FE (304 mg); FF (819 mg); FG (1,351 mg); FH (794 mg); FI (3,239 mg); FJ (384 mg); FK (2,599 mg); FL (1,489 mg); FM (2,029 mg).

From secondary fractions FG and FH (2 g), fargesin (**2**) (607 mg) was isolated after successive CC and the recrystallization procedure with Hex. From secondary fraction FI (3 g), a mixture of fargesin and (8R,8′R,9R)-cubebin (**2** and **3**) was obtained and after successive CC and preparative TLC, 835.9 mg of **3** and 507.7 mg of **2** were purified.

Eupomatenoid-1 (**1**) was obtained as white crystalline needles with an m.p. of 157-158°C (lit, 154–156°C), soluble in CHCl_3_, with a retention time (*R_t_*) = 13.09 min at 220 and 280 nm, and using a Hex : CHCl_3_ 1 : 1 system, it yielded a Retention factor (*R_f_*) = 0.13. IR (KBr): 2,937, 2,849, 1,725, 1,604, 1,493, 1,448, 1,250, 1,142, and 1,041 cm^−1^. IE-MS: *m/z* (rel. int) 322 (100), 295 (10), 291 (10), 202 (15), 121 (6), 77 (5), and 46 (15). ^1^H-NMR (300 MHz, CDCl_3_): 7.03 (1H, d, *J* = 1.5 Hz, H-4), 6.82 (1H, d, *J* = 1.5 Hz, H-6), 7.1 (1H, d, *J* = 2 Hz, H-2′), 7.25–7.32 (1H, d, *J* = 8.2 Hz, H-5′), 6.98 (1H, dd, *J* = 8.2 and 0.6 Hz, H-6′), 6.0 (2H, s, OCH_2_O), 4.03 (3H, s, OCH_3_), 2.40 (3H, s, 3-CH_3_), 6.5 (1H, dd, *J* = 15.6 and 1.5 Hz, H*α*), 6.15–6.27 (1H, dq, *J* = 15.6 and 6.6 Hz, H*β*), and 1.91 (3H, dd, *J* = 6.6 and 1.5 Hz, H-*γ*). ^13^C NMR (75.4 MHz, CDCl_3_): 151.14 (C-2), 110.5 (C-3), 133.0 (C-3a), 133.6 (C-5), 109.2 (C-4), 104.4 (C-6), 177.8 (C-7), 142.1 (C-7a), 123.7 (C-1′), 109.4 (C-2′), 147.4 (C-3′), 147.9 (C-4′), 114.4 (C-5′), 120.6 (C-6′), 101.2 (OCH_2_O), 56.2 (OCH_3_), 9.6 (3-CH_3_), 131.4 (C-*α*), 124.4 (C-*β*), and 18.4 (C-*γ*).

Fargesin (**2**) was obtained as a white powder with an m.p. of 136–139°C (lit, 137–139°C and 133-134°C), soluble in CHCl_3_, with an *R_t_* = 13.52 min. at 220 and 280 nm, and showing *R_f_* = 0.56 with a Hex : EtOAc 1 : 1 system. IR (KBr): 2,960, 2,870, 2,841, 1,606, 1,592, 1,512, 1,492, and 1,240 cm^−1^. IE-MS: *m/z* (rel. int) 370 [M^+^ (100)], 339 (12), 177 (40), 161 (40), 151 (15), 150 (10), 149 (45), 135 (30), and 122 (15). ^1^H-NMR (300 MHz, CDCl_3_): 6.76–6.9 (6H, m, H-2,5,6,2′,5′ and 6′), 4.73 (2H, d, *J* = 4.0 Hz, H-7*α* and 7′*α*), 4.25 (2H, m, H-9*α* and 9*β*) 3.08 (1H, m, 8 and 8′), 3.86 (2H, m, H-9′*α* and 9′*β*), 5.95 (2H, s, OCH_2_O), 3.89 (3H, s, OCH_3_), and 3.86 (3H, s, OCH_3_). ^13^C NMR (75.4 MHz, CDCl_3_): 101.0 (OCH_2_O), 133.6 (C-1), 135.1 (C-1′), 106.5 (C-2), 108.2 (C-2′), 109.3 (C-5), 111.1 (C-5′), 118.2 (C-6), 119.3 (C-6′), 147.1 (C-3), 148.0 (C-3′), 148.7 (C-4), 149.2 (C-4′), 85.3 (7), 85.7 (C-7′), 54.3 (C-8), 71.7 (C-9), 71.7 (C-9′), 54.2 (C-8′), and 56.0 (2 OCH_3_).

(8R,8′R,9R)-cubebin (**3**) was obtained as white needles with an m.p. of 127-128°C, soluble in CHCl_3_, with an *R_t_* = 14.85 min. at 280 nm, and an *R_f_* = 0.37 using a CHCl_3_ system. IR (KBr): 3,365, 2,896, 1,611, 1,492, 1,441, 1,243, and 1,037 cm^−1^. IE-MS: *m/z* (rel. int) 356 (30), 338 (30), 203 (40), 202 (15), 135 (100), and 81 (70). ^1^H-NMR (300 MHz, CDCl_3_): 6.49–6.73 (6H, m, H-2,5,6,2′,5′ and 6′), 5.92 and 5.91 (4H, s, 2 OCH_2_O), 5.22 (1H, d, *J* = 1.5 Hz, H-9*α*), 4.1 (1H, dd, *J* = 8.7, 6.9 Hz, H-9′*α*), 3.78 (1H, dd, *J* = 8.7, 7.2 Hz, H-9′*β*), 2.14 (2H, m, 8′ and 8), 2.43 (2H, m, H-7*α* and 7′*α*), 2.75 (1H, m, H-7*β*), and 2.60 (1H, m, H-7′*β*). ^13^C NMR (75.4 MHz, CDCl_3_): 100.83 and 100.8 (OCH_2_O), 133.2 (C-1), 134.1 (C-1′), 108.0 (C-2), 108.1 (C-2′), 109.15 (C-5), 109.3 (C-5′), 121.7 (C-6), 121.3 (C-6′), 147.6 (C-3), 147.5 (C-3′), 145.8 (C-4), 145.7 (C-4′), 38.4 (C-7), 39.1 (C-7′), 52.2 (C-8), 45.8 (C-8′), 103.3 (C-9), and 72.6 (C-9′).

### 2.4. Test Organisms

*M. tuberculosis* strains H37Rv (ATCC 27294), four monoresistant variants of *M. tuberculosis* H3Rv, including isoniazid-resistant (ATCC 35822), streptomycin-resistant (ATCC 35820), rifampicin-resistant (ATCC 35838), and ethambutol-resistant (ATCC 35798), and two MDR clinical isolates of *M. tuberculosis* (CIBIN/UMF15:99 and SIN 4) were employed as mycobacterium testing organisms. *M. tuberculosis* H37Rv is sensitive to all five first-line antituberculosis drugs (isoniazid, rifampicin, ethambutol, streptomycin, and pyrazinamide), and the two clinical isolates were MDR and resistant to all five first-line antituberculosis drugs. *Entamoeba histolytica* strain HM1-IMSS and *Giardia lamblia* strain IMSS : 0989 : 1 were used as antiprotozoal testing organisms.

### 2.5. Antimycobacterial Activity

The Hex extract and pure compounds were tested using microplate Alamar blue assay (MABA), as previously described [9, 10]. All assays were carried out in triplicate, and isoniazid (0.06 *μ*g mL^−1^, Sigma) and rifampicin (0.062 *μ*g mL^−1^, Sigma) were included as positive control drugs to H37Rv-sensitive strains. For MDR *M. tuberculosis* (CIBIN/UMF15:99 and SIN 4), isoniazid and rifampicin were employed at 3.13 and 100.0 *μ*g mL^−1^, respectively. Ofloxacin at 0.5–16 *μ*g mL^−1^ was also used as a positive control by CIBIN/UMF15:99. Antimycobacterial activity was reported as the Minimal Inhibitory Concentration (MIC).

### 2.6. Antiprotozoal Activity

*E. histolytica* strain HM1-IMSS was cultured in a TYI-S-33-modified medium supplemented with 10% calf serum, and *G. lamblia* strain IMSS : 0989 :  1 was maintained in a TYI-S-33 medium supplemented with 10% calf serum and bovine bile. *In vitro* susceptibility assays for both strains were performed by using the method previously described [11, 12]. Briefly, 5 × 10^4^ trophozoites of* G. lamblia *were incubated for 48 h at 37°C with increasing concentrations of the Hex extract of *A. elegans *and the purified compounds. After incubation,* G. lamblia *trophozoites were washed and subcultured for an additional 48 h in fresh medium alone. For *E. histolytica,* 6 × 10^3^ trophozoites were incubated for 72 h at 37°C with increasing concentrations of the samples tested. Dimethyl sulfoxide (DMSO) was used as a suitable solvent. Albendazole and metronidazole were included as positive controls; parasites without treatment were included as a negative control.* G. lamblia *and* E. histolytica *trophozoites were counted, and the 50% Inhibitory Concentration (IC_50_) was calculated by Probit analysis. Experiments were carried out in triplicate and repeated at least twice. Eupomatenoid-1 was also evaluated against *Trichomonas vaginalis *strain GT9 following the same procedure as for* E. histolytica.*

## 3. Results

### 3.1. Chemical Characterization of the Purified Compounds

**Figure 1:**
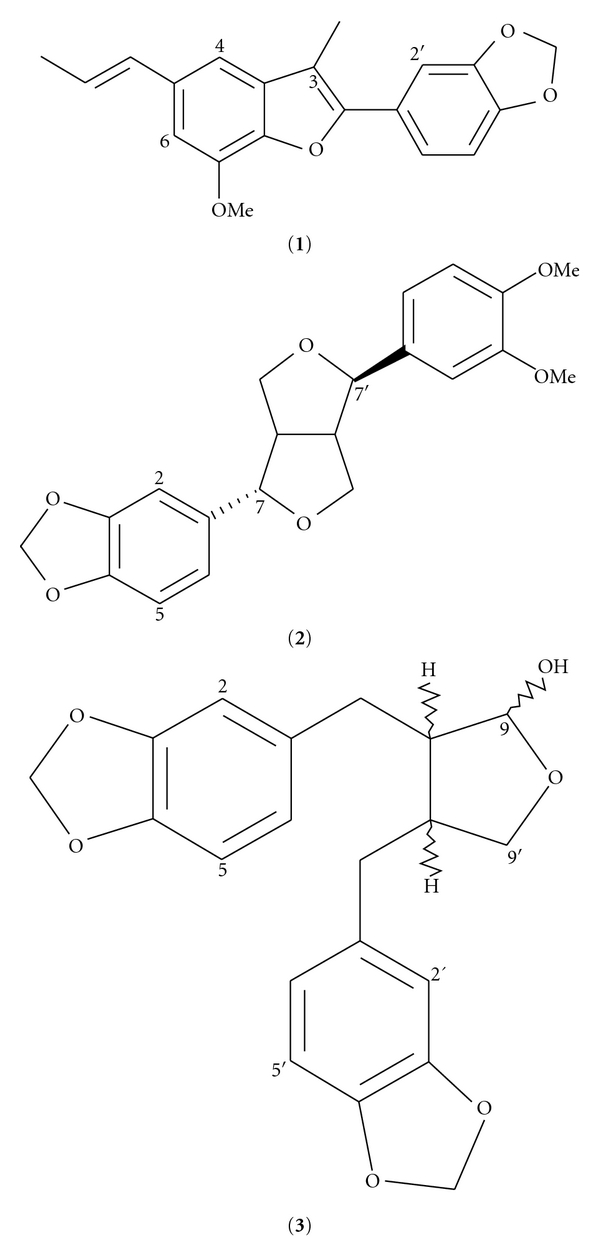
Chemical structures of isolated compounds from *A. elegans* hexanic extract.

In this study, we describe the isolation of eupomatenoid-1 (**1**), fargesin (**2**), and (8R,8′R,9R)-cubebin (**3**) (Figure 1) from the Hex extract of *A. elegans* rhizomes by chemical fractionation on CC. Their structures were elucidated according to ^1^H-NMR, ^13^C-NMR, and MS data and were in agreement with those previously described in the literature. In the HPLC analysis, the eupomatenoid-1 showed an *R_t_* = 13.09 min. using acetonitrile/formic acid 98 : 2 system, while fargesin and (8R,8′R,9R)-cubebin showed  *R_t_* = 13.52 and 14.85 min., respectively, when MeOH was employed; all compounds were detected at 220 and 280 nm.

### 3.2. Antimycobacterial and Antiprotozoal Evaluation

The antimycobacterial activity of the Hex extract and purified compounds determined by the MABA is depicted in Table 1. Although Hex extract and eupomatenoid-1 were inactive against *M. tuberculosis* H37Rv (MIC > 100 *μ*g mL^−1^), fargesin and (8R,8′R,9R)-cubebin exhibited good activity against this strain (MIC = 50 *μ*g mL^−1^). It is noteworthy that the Hex extract and compound **3** were active against the two MDR *M. tuberculosis* clinical isolates: CIBIN/UMF15 : 99, and SIN4 (MIC = 50 *μ*g mL^−1^), while compound **2 **inhibited only the growth of SIN4 (MIC = 50 *μ*g mL^−1^). In addition, compound **2 **was the most active against the monoresistant variants of *M. tuberculosis* H37Rv (MIC = 12.5–25 *μ*g mL^−1^) with the exception of the ethambutol-resistant strain (MIC > 50 *μ*g mL^−1^). Compounds **1 **and **3 **were moderately active against all monoresistant strains of *M. tuberculosis* H37Rv tested (MIC = 100 *μ*g mL^−1^).

The antiprotozoal activity of the Hex extract and of pure compounds **1**–**3** was tested against the anaerobic protozoa *E. histolytica* and *G. lamblia *(Table 1). It was observed that the Hex extract was active against these two parasites, exhibiting IC_50_ = 0.235 and 0.315 *μ*g mL^−1^, respectively. On the other hand, compound **1 **was the most active compound against *E. histolytica* and *G. lamblia*, achieving IC_50_ values of 0.624 and 0.545 *μ*g mL^−1^, respectively. Compounds **2 **and **3 **demonstrated moderate antiprotozoal activity with IC_50_ < 275.00 *μ*g mL^−1^ against both parasites. Because of its important antiprotozoal activity, eupomatenoid-1 was evaluated against *T. vaginalis,* showing an IC_50_ = 0.840 *μ*g mL^−1^. 

## 4. Discussion

The presence of the lignans and neolignans in *A. elegans* has been described [2, 5]; however, in this study the presence of eupomatenoid-1 (neolignan), fargesin, and (8R,8′R,9R)-cubebin (lignans) has been described for the first time in *A. elegans* rhizome. In this work, the analytical conditions that can be employed for detecting these compounds are also described.

Compound **1 **has previously been isolated from *Eupomatia laurina*, *A. taliscana*, and *Caryodaphnosis baviensis*, and a related compound, such as eupomatenoid-7, has been found in *A. taliscana *[13–17]. Compound **2 **has been isolated from *Horsfieldia iryaghedhi* (*Myristica horsfieldia*), *Piper sarmentosum*, *Magnolia biondii*, *Stauranthus perforatus*, and *Aristolochia malmeana *[18–23]. Compound **3 **has been isolated from related species such as *A. legasiana*, *A. malmeana*, *A. odoratissima*, and *A. pubescens* [21, 22, 24]. In fact, structurally similar compounds such as aristelegin A-C have been reported for the roots and stems of *A. elegans* [5]. 

Of the three pure compounds, fargesin (**2**) was the most active against the mycobacterium strains tested (MIC < 50 *μ*g mL^−1^); compound **3 **showed activity against *M. tuberculosis* H37Rv and two MDR strains of *M. tuberculosis*. Eupomatenoid-1 (**1**) was slightly active against *M. tuberculosis* H37Rv, its monoresistant variants and two MDR *M. tuberculosis* clinical isolates, in comparison with eupomatenoid-7, a compound structurally similar to eupomatenoid-1, that we have previously demonstrated to be more active against the same strains with MIC values <25 *μ*g mL^−1^ [16]. These data suggest that the methylenedioxy group in the eupomatenoid-1 molecule exerts a negative influence on its antimycobacterial activity, since eupomatenoid-7 does not possess this group and was more active against several mycobacterium strains; nevertheless, further structure-activity studies are needed to confirm this hypothesis.

It is noteworthy that fargesin was active against *M. tuberculosis* H37Rv, its monoresistant strains, and to a lesser degree against the MDR SIN4 isolate (MIC < 50 *μ*g mL^−1^); on the other hand some related compounds such as (+)-sesamin and horsfieldin (isolated from *Piper sarmentosum*) were inactive against the *M. tuberculosis* H37Rv strain (MIC > 200 *μ*g mL^−1^) [25]. The bacteriostatic activity of (8R,8′R,9R)-cubebin has been reported against *Streptococcus mitis, Enterococcus faecalis*, *Ostrinia nubilalis,* and *Anticarsia gemmatalis* [21, 24–27]. Interestingly, in this study it has been demonstrated that compound **3** was active against the two MDR *M. tuberculosis* clinical isolates tested showing a MIC value of 50 *μ*g mL^−1^. Our data suggest that compounds **2** and **3** are two of the possible compounds responsible for the antimycobacterial activity exerted by the Hex extract of *A. elegans*-rhizome.

Current tuberculosis chemotherapy is prolonged (24 months), poorly effective, expensive, and is accompanied by severe side effects. Besides, the presence of MDR *M. tuberculosis* cases is rapidly increasing. MDR accounts for 5.3% of all TB cases reported around the world [28, 29], underlining the importance of using new alternatives in the treatment of tuberculosis. In this regard, medicinal plants have proven to be an important source of antimycobacterial compounds [28, 30–32]. In fact, it was demonstrated that purified compounds **2 **and **3** showed significant activity against monoresistant and MDR *M. tuberculosis* strains.

A murine model of tuberculosis previously developed by Hernández-Pando et al. [33] could be further used to determine the *in vivo* activity of compounds **2 **and **3**, resulting in insights concerning their potential as antitubercular agents. On the other hand, the chemical structure of these compounds can be a prototype for the design and synthesis of new derivatives with enhanced antimycobacterial activity.

*G. lamblia* and *E. histolytica *are two of the most clinically important anaerobic protozoa that cause diarrheal disease worldwide. Recently, giardiasis was included in the “Neglected Disease Initiative”, estimating that 280 million people are infected each year with *G. lamblia *[34]. Therefore, this stimulated our interest in determining the potential activity of the Hex extract of *A. elegans*-rhizome and its purified compounds against these two protozoa. Metronidazole was included as a reference drug because it has been regarded as the choice drug for the treatment of giardiasis and amoebiasis, although it is not always effective and has severe side effects. 

The Hex extract and eupomatenoid-1 were the most active against both *E. histolytica *and* G. lamblia*. It should be mentioned that metronidazole was just 1.4 and 4 times more potent than the Hex extract and 2.5 and 10 times more active than eupomatenoid-1, respectively. The antiprotozoal activity of eupomatenoid-1 needs to be supported by a demonstration of its efficacy in animal models as well as by a clear understanding of its action mechanisms.

Several studies supporting the use of natural products and their purified active compounds are an alternative treatment for gastrointestinal infections. In particular, the antiprotozoal activity of *Helianthemum glomeratum* Lag. and *Rubus coriifolius* Focke was demonstrated *in vitro *and* in vivo* [35, 36]. The *in vitro* activity of MeOH extract from *H. glomeratum* and *R. coriifolius* showed IC_50_ = 62.92 and 77.82 *μ*g mL^−1^ against *G. lamblia*; in addition, in a mouse model of giardiasis, these extracts showed an ED_50_ = 0.125 and 0.506 mg kg^−1^, respectively [36]. The most active compound isolated from these plants was (−)-epicatechin, this compound showed an *in vitro* IC_50_ = 1.6 *μ*g mL^−1^ against *G. lamblia *and in a mouse model of giardiasis had an ED_50_ = 0.072 *μ*mol kg^−1^.

The inappropriate short-term exposure and exposure to sublethal levels of metronidazole have induced parasite drug resistance. Eupomatenoid-1 may therefore be considered as an active principle or even a prototype molecule for the development of novel antiprotozoal agents with activity against metronidazole resistant parasites.

## 5. Conclusion

In this study, the activity of (8R,8′R,9R)-cubebin and fargesin, purified from the Hex extract of *A. elegans*-rhizome, was demonstrated against *M. tuberculosis* H37Rv, four monoresistant variants, and two MDR *M. tuberculosis* clinical isolates. Although eupomatenoid-1 showed poor antimycobacterial activity, it had significant antiprotozoal activity. These active compounds can be prototype molecules for the design and synthesis of new derivatives with enhanced antimycobacterial or antiprotozoal activity.

Is currently being evaluated, the acute and subacute toxicity of active compounds in a mouse model. Further *in vivo* studies may well support the antimycobacterial and antiprotozoal activities of *A. elegans-*rhizome purified compounds.

The antiprotozoal activity of neolignans and lignans has scarcely been described in the literature, and our results encourage further studies on this issue.

## Figures and Tables

**Table 1:** Antimycobacterial and antiprotozoal activities of the hexanic extract and pure compounds isolated from *A. elegans. *

Sample	MIC (*μ*g mL^−1^) *M. tuberculosis *							IC_50_ (*μ*g mL^−1^)	
	H37Rv	CIBIN/UMF15 : 99	SIN4	RIF-R	STR-R	INH-R	EMB-R	*E. histolytica*	*G. lamblia*
Hexanic extract	>100	50	50	ND	ND	ND	ND	0.235	0.315
Eupomatenoid-1	100	100	100	100	100	100	100	0.624	0.545
Fargesin	50	>100	50	25	25	12.5	>50	120.6	262.7
(8R,8′R,9R)-Cubebin	50	50	50	100	100	100	100	137.3	275.0
Rifampicin	0.06	>100	100	>25	0.06	0.06	0.06	—	—
Isoniazid	0.06	3.1	3.1	0.06	0.06	>25	0.06	—	—
Streptomycin	0.5	>100	>4	0.5	>8	0.5	0.5	—	—
Ethambutol	2.0	8	>16	1.0	1.0	1.0	>32	—	—
Ofloxacin	—	0.5	8.0	—	—	—	—	—	—
Metronidazole	—	—	—	—	—	—	—	0.060	0.210

H37Rv: sensitive strain to INH, RIF, EMB, STR, and pyrazinamide; CIBIN/UMF15:99: resistant strain to INH, RIF, EMB, STR, and pyrazinamide; SIN4: resistant strain to INH, RIF, EMB, STR, rifabutin, ethionamide, and ofloxacin; RIF-R: rifampicin-resistant; STR-R: streptomycin-resistant; INH-R: isoniazid-resistant and EMB-R: ethambutol-resistant. ND: no determined; MIC: minimum inhibitory concentration; IC_50_: 50% inhibitory concentration. Data are means of three determinations.
